# Comparison of Three Diagnostic Methods to Detect the Occurrence of *Fasciola* Species in Communally Grazed Cattle in the North West Province, South Africa

**DOI:** 10.3390/pathogens11121398

**Published:** 2022-11-23

**Authors:** Sunday C. Olaogun, Charles Byaruhanga, Sunday O. Ochai, Geoffrey T. Fosgate, Munyaradzi C. Marufu

**Affiliations:** 1 Department of Production Animal Studies, University of Pretoria, Private Bag X4, Onderstepoort, Pretoria 0110, South Africa; 2 Department of Veterinary Medicine, Faculty of Veterinary Medicine, University of Ibadan, Ibadan 200005, Nigeria; 3 Department of Veterinary Tropical Diseases, University of Pretoria, Private Bag X4, Onderstepoort, Pretoria 0110, South Africa

**Keywords:** antigen ELISA, bovine, *Fasciola* species, real-time PCR, sedimentation

## Abstract

Fasciolosis causes significant economic losses in commercial cattle herds in South Africa, but its prevalence is unknown in most communal areas. A cross-sectional study was conducted with the aim of determining the occurrence of bovine fasciolosis using three different diagnostic methods in Moretele Local Municipality in Bojanala District, North West Province. Faecal samples were collected from 277 cattle of different breeds, ages, sex and faecal condition scores and examined using the sedimentation technique, quantitative real-time polymerase chain reaction (qPCR) and faecal antigen enzyme-linked immunosorbent assay (coproELISA). All samples were negative for bovine fasciolosis using coproELISA. A total of 73 (26.4%) samples were positive using the qPCR, while 36 were positive using the sedimentation technique, with low faecal egg counts (1 to 20 eggs per gram). The qPCR detected the highest positivity (26.4%, 95% CI 21.3, 32.0) followed by the sedimentation test (13.0%; 95% CI 9.3, 17.5). Location, breed, sex, age and faecal consistency score were not associated with positive qPCR results (*p* > 0.05). There was also no significant agreement (kappa = −0.011, *p* = 0.843) between qPCR and the sedimentation technique for the detection of *Fasciola* spp. The qPCR appeared to be the most sensitive method for detection of *Fasciola* spp. Further studies are required on the characterisation of *Fasciola* spp. in communal cattle in South Africa.

## 1. Introduction

Fasciolosis, also known as distomatosis or liver fluke disease, is an important neglected parasitic zoonosis caused by trematodes of the genus *Fasciola* (Phylum Platyhelminthes: Family Fasciolidae) [1,2]. The most common species are *F. hepatica* in temperate regions and *F. gigantica* in tropical countries [3,4,5]. Hybrids from both *F. hepatica* and *F. gigantica* have also been reported in some countries including South Africa [5,6,7,8]. Transmission of *Fasciola* spp. is by freshwater snails of the family Lymnaeidae [9]. In South Africa, *F. hepatica* is mainly transmitted by *Galba truncatula* [10], while *F. gigantica* is mainly transmitted by *Radix natalensis* [11]. *Pseudosuccinea columella* is capable of transmitting both *Fasciola* spp. [12]. The disease is widely distributed globally and affects humans and a wide range of wild and domestic ruminants [13,14], causing severe losses.

Economic losses affect cattle farmers, butchers and consumers in the form of liver condemnation, reduction in growth rate, poor carcass quality, poor conception rate and mortality [15,16]. Annual productivity losses due to fasciolosis in livestock have been estimated to exceed USD 302 billion, while global annual economic losses exceed USD 200 million [16]. In South Africa, annual financial losses of ZAR 129, 901 (USD 9992.40) were estimated due to whole liver condemnation among slaughter cattle in the Eastern Cape Province [17]. These losses necessitate evidence-based information about occurrence of the diseases so as to implement effective mitigation strategies. Few studies have determined the prevalence of fasciolosis in South Africa and these have been largely abattoir-based (postmortem diagnosis) and biased towards commercial cattle production. An on-station study by Ndlovu et al. [18] demonstrated a *Fasciola* spp. prevalence of 16.3% in cattle on a research farm in the Eastern Cape Province, using the formalin-ether sedimentation method. In another study, Jaja et al. [17] reported the highest prevalence of fasciolosis in summer (23%) and the lowest (5%) in winter in slaughter cattle in the same province using post mortem liver inspection. Recently, Mpisana et al. [19] reported an overall prevalence of 39.1% in slaughtered dairy cattle in the Eastern Cape Province using liver inspection. There is still scant data from antemortem studies about the occurrence of bovine fasciolosis, especially in communal areas across the country, where farmers have little knowledge about the disease.

Antemortem detection of *Fasciola* spp. in cattle has traditionally been achieved using the sedimentation method for faecal egg counts (FEC), or by using the faecal antigen enzyme-linked immunosorbent assay (coproELISA). The sedimentation method detects eggs of *Fasciola* spp. in faeces from patent infections and not pre-patent infections. Furthermore, the method is laborious, does not differentiate between the different *Fasciola* spp. and has low sensitivity [20]. The coproELISA, on the other hand, can detect *Fasciola* spp. secretory–excretory antigens in faeces even during the pre-patent period, but also cannot differentiate between *Fasciola* spp. Moreover, the method has low sensitivity when FECs are low [20,21]. A quantitative real-time polymerase chain reaction (qPCR) assay was developed and this can detect *Fasciola* DNA in faecal samples even with very low egg counts [22] and as early as two weeks post-infection [23,24]. Given the potential impact of fasciolosis, there is need to evaluate and compare the performance of different diagnostic methods so as to effectively determine disease occurrence in herds and to implement appropriate control strategies.

The North West Province has approximately 1,776,000 beef cattle, which is about 12.8% of the cattle population of South Africa [25]. To date, there is no published report about the occurrence of the disease among cattle owned by smallholder farmers in this province. Smallholder farmers in this area have limited knowledge about disease occurrence and there is less likelihood of implementing appropriate prevention and control practices towards bovine fasciolosis (Olaogun, unpublished data).

The objectives of the current study were, therefore, to estimate the prevalence of fasciolosis in naturally infected cattle reared communally by smallholder farmers in the North West Province, South Africa using the sedimentation technique, qPCR and coproELISA, and to compare the detection rate across the three methods. It was hypothesised that the qPCR would detect a higher prevalence compared to coproELISA or sedimentation.

## 2. Materials and Methods

### 2.1. Ethical Approval

Ethical clearance for this study was obtained from the Faculty of Veterinary Science Research Ethics Committee (REC0158-19) and Animal Ethics Committee (REC 158-19) at the University of Pretoria, South Africa. Permission to conduct research under Section 20 of the Animal Diseases Act (Act 35 of 1984) was provided by the Department of Agriculture, Land Reform and Rural Development of the Republic of South Africa (12/11/1/18).

### 2.2. Study Area

**Figure 1:**
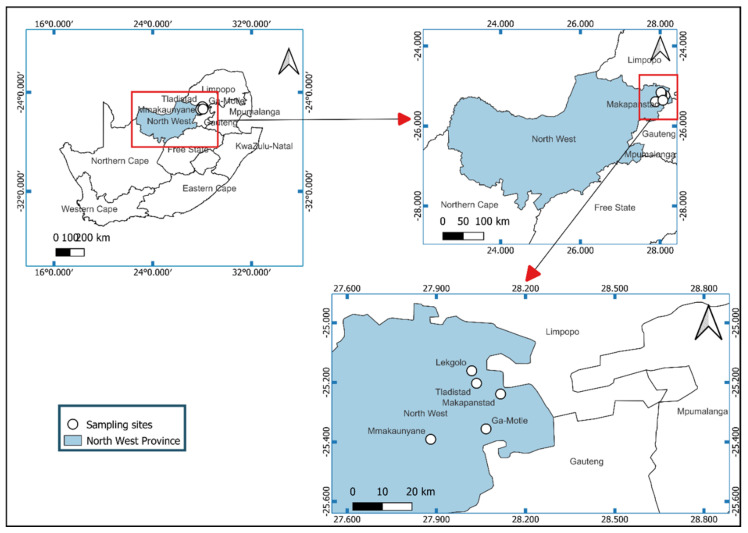
A map showing the study locations (marked in white dots) in Moratele Local Municipality in the, North West Province, South Africa.

The study was conducted in Makapanstad, Lekgolo, Tladistad, Mmakaunyane and GaMotle villages of Moretele Local Municipality (administered under Bojanala District Municipality) in the North West Province of South Africa. The North West is one of the nine provinces of South Africa and is located in the northwestern part of the country (Figure 1). Makapanstad village covers an area of 20.45 km^2^ with a total human population of 15,076. Its geographical coordinates are latitude 25°14′36″ S and longitude 28°7′19″ E. Ga-Motle is a populated place in Bojanala District Municipality. It is located at an elevation of 1068 m above sea level. Its coordinates are 25°21′0″ S and 28°4′0″ E. The annual rainfall of these areas is 600 mm, received mostly in summer (November–March), and the temperature averages 12 °C in the winter season and 25 °C in summer [26].

### 2.3. Study Design and Sample Size

The study district, local municipality and the five villages were selected based on ease of access to communally farmed cattle herds as well as farmers’ willingness and commitment to participate in the study. From the five villages, a total of 50 herds were randomly sampled from a sampling frame provided by the veterinary authorities. A sample size of 275 was estimated using the formula by Thrusfield et al. [27] as follows:

$$n=\frac{z_{1-\alpha /2\;}^{2}P\left(1-P\right)}{d^{2}}$$

where *n* = required sample size; $z_{1-\alpha /2}$ is the z-score for the desired level of confidence (95%); *d* = absolute precision (5%); *P* = expected prevalence. An estimated prevalence of 23.3% was used based on a previous study on detection of *Fasciola* spp. by post mortem liver inspection at abattoirs in the Eastern Cape Province of South Africa [17]. Cattle in each herd were selected by a systematic random sampling method. This started with selection of an animal in a herd at random and then every H^th^ animal, where H was the sampling interval determined by dividing the herd size by the desired sample size. On average, five heads of cattle were sampled in each herd.

### 2.4. Collection of Faecal Samples and Animal Records

Faecal samples were collected by means of a lubricated gloved hand from the rectum of each animal into 80 mL plastic faecal containers, which were subsequently labelled with the village name, date of sampling, breed, sex, age and faecal score. The age was determined using dentition and information from the farmers, and the animals were categorised as 2–4 years (young adults) or above 4 years (adults). Breed type was determined based on phenotypic characteristics as previously described [28,29], and this was complemented by farmer records. Faecal samples were scored as poor, average and good based on previously described criteria [30]. The collected samples were placed in a cooler box with ice packs, and then transported to the parasitology laboratory in the Department of Veterinary Tropical Diseases at the University of Pretoria, South Africa for further analysis.

### 2.5. Sedimentation Technique to Identify Fluke Eggs

Eggs of *Fasciola* spp. were identified and enumerated using a sedimentation technique as described by Calvani et al. [22] with some modifications. Six grams of faeces were homogenised with 20 mL of distilled water in an 80 mL plastic container using a wooden spatula. The mixture was filtered using distilled water from a high-pressure source into a 95 µm filter placed in another 50 µm filter. The inward filter was rinsed and withdrawn. The residue from the 50 µm filter was collected into a 1000 mL glass beaker by rinsing with water. The beaker was filled with water and left to stand for 5 min. The supernatant was decanted, followed by refilling the beaker with distilled water and being left to stand for another 5 min. After the second rinsing, most of the supernatant was decanted and the remaining mixture was poured into a measuring cylinder (of capacity 100 mL) and left to stand for 5 min. The supernatant was again decanted to leave a sediment of about 10 mL and two drops of methylene blue (1%) were added to the sediment and mixed by shaking. Examination for *Fasciola* eggs was performed under a light microscope at 10× magnification (Olympus microscope, New York microscope company, Hicksville, NY, USA). The number of *Fasciola* eggs in all grids in the counting chamber were counted and recorded. Happich [31] demonstrated that about one third of eggs from the initial faecal sample volume are retained in the final processed sediment. Therefore, the value of eggs per gram (EPG) for each faecal sample was calculated by multiplying the number of eggs counted by 3 and then dividing this by 6 (the initial grams of faeces). The 10 mL sediment was collected in a sterile plastic container for subsequent DNA extraction, which was conducted on the same day.

### 2.6. DNA Extraction

DNA was extracted from the faecal sediments (*n* = 277) using the QIAamp^®^ Fast DNA Stool Mini Kit (QIAGEN, Hilden, Germany) as described by Calvani et al. [22], with some modifications. The faecal sediment was poured into a 15 mL plastic cylindrical tube and centrifuged at 4000 rpm for 40 min. The pellet was collected using a sterile fine wooden applicator stick and placed into a pre-prepared tube containing ceramic beads (MagNA Lyser Green Beads, Roche Diagnostics, Mannheim, Germany) and 700 µL of Lysis buffer, AL (QIAGEN, Hilden, Germany). The mixture was homogenised at 6800 revolutions per second for 36 s in a Precellys 24 Tissue Homogeniser (Bertin Technologies SAS, Montigny-le-Bretonneux, France). This was followed by incubation at 85 °C for 10 min. About 600 µL of the mixture was transferred into a 2 mL microcentrifuge tube containing 25 µL proteinase K, followed by addition of 700 µL of InhibEx from the extraction kit. The mixture was vortexed for 15 s and then incubated at 70 °C for 24 h. The rest of the DNA extraction procedure, starting with the addition of ethanol, was as described in the QIAamp^®^ Fast DNA Stool Mini Kit protocol. The extracted DNA was stored at −20 °C until further analysis.

### 2.7. Quantitative Real-Time PCR for Fasciola Species

A previously published qPCR assay was used to amplify a 140-base pair (bp) fragment of the *Fasciola* internal transcribed spacer 2 (*ITS-2*) gene from the faecal samples. The PCR primers and probes as well as the procedure used in this study were as previously described [32], with some modifications. The *F*. *hepatica* (ProFh) and *F*. *gigantica* (ProFg) probes were instead labelled with FAM or VIC reporter dyes, respectively, at the 5′ ends and each probe labelled with a QSY quencher dye at the 3′ end. Each PCR reaction comprised 1X TaqMan^®^ Universal PCR Master Mix (Applied Biosystems, Life Technologies, Johannesburg, South Africa), 0.3 μM of each primer, 0.1 μM of the FAM- and VIC-labelled probes and 2 μL of DNA template in a total reaction volume of 20 μL. Thermal cycling was done in a StepOnePlus™ Real-Time PCR System (Applied Biosystems, Life Technologies, Johannesburg, South Africa) under the following conditions: Uracil N-Glycosylase digest at 50 °C for 2 min, followed by AmpliTaq Gold pre-activation at 95 °C for 10 min and then 45 cycles of amplification at 95 °C for 15 s and annealing at 60 °C for 1 min. Positive controls were DNA previously extracted from adult *Fasciola* worms obtained from infected livers, confirmed using PCR and DNA sequencing. The negative control was nuclease-free water.

### 2.8. Coproantigen ELISA (coproELISA)

The *F. hepatica* MONOSCREEN antigen indirect Sandwich ELISA kit (Bio-X Diagnostics, Rochefort, Belgium) was used for the detection of trematode coproantigens, following a procedure described by the manufacturer. Rows A, C, E and G of the microtiter plate were coated with a polyclonal antibody that is specific to *F. hepatica*, while rows B, D, F and H were coated with a polyclonal antibody MM3 that is not specific to the parasite. This was to allow differentiation between a specific immunological reaction and nonspecific binding. Two grams of faecal material were diluted in 2 mL of dilution buffer and then centrifuged at 1000 g for 10 min. The supernatant was collected and 100 µL of this was added to the corresponding microplate wells. Samples were added as follows: sample 1 in wells A1 and B1, and the other samples and controls were added in that order. The plates were incubated at 24 °C for 2 h on a rotatory incubator (Environmental Shaker-Incubator ES-20, Biosan Ltd., Riga, Kurzeme, Latvia). Afterwards, the plates were washed three times with the wash buffer, followed by addition of 100 µL of the diluted biotin-linked anti-*F. hepatica* conjugate to each well. The plates were again incubated at 24 °C for 1 h on the rotatory incubator and then washed three times. Following this wash step, 100 µL of the avidine–peroxidase conjugate was added to each well and incubated at 24 °C for 1 h. Subsequently, the plates were washed three times and 100 µL of the chromogen was added, followed by incubation in the dark at room temperature for 10 min. This was followed by the addition of 50 µL of stop solution to each well. The optical densities (OD) in the microwells were read at 450 nm using a microplate reader (BioTek Power Wave XS2microplate reader, Agilent Technologies Inc, Santa Clara, CA, USA) immediately after the addition of the stop solution. The net OD of each sample was calculated by subtracting from the reading for each sample well (A, C, E, G) the optical density of the corresponding negative control (B, D, F, and H). The net OD of the positive control antigen was calculated in the same way. The change in OD for each sample well was divided by the corresponding positive control signal and multiplied by 100 to express the result as a percentage of the positive control. Samples with values greater than 8% were considered positive.

### 2.9. Data Analysis

Descriptive statistics were performed to determine the distribution of FEC (expressed as eggs per gram) and quantification cycles (Cq) from the sedimentation technique and qPCR, respectively. The sedimentation technique was considered as the gold standard for occurrence of *Fasciola* spp. Apparent prevalence and corresponding 95% confidence intervals were calculated based on the proportion of positive results on each diagnostic assay. Univariate analysis was used to estimate the association between qPCR status for *Fasciola* spp. (positive, negative) and potential risk factors (location, breed, age, sex) using chi-square tests. A generalised linear model was used to determine the association between faecal consistencies—categorised as poor, average and good [30]—with the presence of *Fasciola* spp. DNA (qPCR determined). The Cohen’s Kappa test was used to determine the level of agreement across three methods (sedimentation, qPCR, coproELISA). The kappa value was categorised as poor ( <0.00), slight (0.00–0.20), fair (0.21–0.40), moderate (0.41–0.60), substantial (0.61–0.80) and almost perfect (0.81–1.00) as previously described [33]. Data were analysed using R software version 4.0.5 [34] and results were interpreted at the 5% significance level.

## 3. Results

### 3.1. Description of the Sampled Cattle

A total of 277 cattle were sampled for faeces in five locations as follows; Legkolo (*n* = 50), GaMotle (*n* = 60), Makayauna (*n* = 65), Tladistad (*n* = 55) and Makapanstad (*n* = 47). An average of five cattle were sampled per herd and a maximum of 10 herds were sampled per village. Most cattle were nondescript crossbreds (*n* = 143), followed by Brahman (*n* = 90) and Afrikaner (*n* = 32) breeds (Table 1). Other breeds were: Bonsmara (*n* = 4), Boran (*n* = 1), Charelac (*n*-1), Limosine (*n* = 2), Nguni (*n* = 3) and Simental (*n* = 1). A higher number of sampled cattle (*n* = 153) were 2 to 4 years of age compared to those above 4 years old (*n* = 124). Fewer males (*n* = 31) than females (*n* = 246) were sampled.

### 3.2. Presence of Fasciola Eggs in Faecal Samples Using the Sedimentation Technique

The sedimentation technique detected eggs of *Fasciola* spp. in 36 (13.0%; 95% CI 9.3, 17.5) of the total 277 bovine faecal samples examined. The FEC from the samples were generally low; of the 36 positive samples, 30 (83%) had 1 to 4 EPG, while 3 samples (8%) had 5 to 10 EPG and another 3 samples had >10 EPG.

### 3.3. Occurrence of Fasciola Species in Faecal Samples Using qPCR

Of the 277 cattle sampled, 73 (26.4%, 95% CI 21.26, 31.96) were positive for *Fasciola* spp. using the qPCR assay. More than half of the qPCR positive samples (*n* = 41) showed quantification cycle (Cq) values greater than 30, while 14 samples had Cq values of 26 to 30, and only 18 samples showed Cq values less than 25. Of the four variables (location, breed, age, sex) assessed in univariate analyses, only location (*p* < 0.001) and age (*p* = 0.045) were significantly associated with detection of *Fasciola* spp. (Table 1). Multivariable analysis did not identify any significant predictor or effect on infection with *Fasciola* spp.

### 3.4. Association between Faecal Consistency and Presence of Fasciola DNA in Faeces

About three-quarters (*n* = 206) of the collected faecal samples were of average consistency, while 41 were of good consistency and 30 were in the poor category. Results of a generalised linear model showed that there was no statistically significant association (*p* > 0.05) between positivity for *Fasciola* spp. and faecal consistency (Table 2). However, faecal samples with poor (30.0%) or average (27.7%) consistency were about two times more likely to be positive for *Fasciola* spp. compared to samples with good (17.1%) consistency (Table 2).

### 3.5. Comparison of Detection Rate of Fasciola spp. between Sedimentation and qPCR

There was no agreement above chance (kappa = −0.011, *p* = 0.843) in the detection of *Fasciola* spp. between the qPCR and the sedimentation technique. The sensitivity of the qPCR assay was 25.0% and specificity was 73.4%, considering the sedimentation method as the gold standard (Table 3). Of the 277 samples tested, 23.5% (*n* = 64) were positive with qPCR but negative with sedimentation, while 9.7% (*n* = 27) were positive with sedimentation but negative with qPCR. Only nine (3.2%) were positive with both sedimentation and qPCR (Table 3).

### 3.6. Detection of Fasciola spp. Using coproELISA

A total of 204 faecal samples (from the overall 277 samples) were tested for the *Fasciola* antigen using a Ag ELISA kit. All the 204 samples tested negative with the coproELISA.

## 4. Discussion

Three diagnostic tests were used in the present study to detect the occurrence of *Fasciola* spp. in naturally infected cattle belonging to smallholder farmers in five villages in the North West Province of South Africa with the aim of establishing the most suitable diagnostic method for on-farm testing of the occurrence of bovine fasciolosis. Calvani et al. [22] previously demonstrated good correlation between qPCR diagnostic workflow and sedimentation in detecting experimental *Fasciola* spp. infection in cattle in Laos. It was not known how this relationship would be influenced by natural infection of cattle under field conditions or how it would compare to detection by the coproELISA method. The current study is, to the authors’ best knowledge, the first to compare the detection of *Fasciola* spp. by the three tests in naturally infected cattle under field conditions in South Africa.

There was an observed variation in detection rate by the three diagnostic methods in the present study. A larger proportion of samples detected positive for *Fasciola* spp. using qPCR than the sedimentation method, and none of the samples detected positive using the coproELISA. Our findings are in agreement with those of Calvani et al. [35], who observed a higher detection rate using qPCR compared to the sedimentation method in sheep. The qPCR assay has been previously reported to be the most sensitive diagnostic method [23,35].

The qPCR produced a greater percentage positivity (26.4%) for *Fasciola* spp. from the faecal samples than the sedimentation method. However, the present study’s qPCR sensitivity of 25.0% from cattle samples was much lower than the 91 to 100% reported previously from sheep faecal samples [35]. This can be explained by the very low faecal egg counts (1 to 4 EPG in 83% of positive samples) in the current study compared with the relatively high infection rate in the study by Calvani et al. [35]. These authors reported up to 267 EPG in faecal samples, an 80% infection rate for *F*. *hepatica* and an average of 61 EPG. This may suggest that sensitivity of the qPCR-based molecular workflow decreases in sub-clinical or chronic infections. Indeed, Calvani et al. [35] demonstrated that DNA amplification by real-time PCR is associated with faecal egg load. Furthermore, the qPCR-based molecular workflow may not yield positive results at ≤2 weeks post-infection [35]. Though two separate probes for *F. hepatica* and *F. gigantica* were used as either individual probes or duplex, they all detected the same 73 samples as positive. This could likely be an indication of mixed *Fasciola* spp. infections (including hybrids) within the positive samples or might indicate poor specificity of the qPCR to differentiate between the species in faecal samples. The later assertion is concerning as the qPCR was reported to differentiate between *F. hepatica* and *F. gigantica* DNA [32]. There is need to develop and evaluate new *Fasciola* qPCR probes that can differentiate between the two species. Calvani et al. [36] developed single nucleotide polymorphism (SNP) assays targeting the *ITS1* and 1s RNA genes of *Fasciola* spp. and confirmed the identity of the infecting *Fasciola* spp. using Illumina sequencing to diagnose infections in faecal samples. To accurately diagnose and differentiate between the two *Fasciola* spp. in naturally infected cattle, there is a need to employ further molecular tools such as cloning and sequencing in conjunction with the qPCR. This can be supported by surveillance data from the abattoirs, in which the flukes recovered from the bile duct at post mortem inspection can accurately be identified to the species level.

Caution needs to be taken when interpreting results from cattle herds that have generally low egg counts as it has been shown that the sedimentation/filtering process has an inherent unintended effect of losing or overlooking eggs, which might reduce the sensitivity of the test at the lower detection limit [37]. False negative FECs have been reported in field studies [38,39] and these could be indicative of pre-patent infections or irregular shedding patterns of *Fasciola* eggs via the biliary system in cattle [20]. Duplicate sedimentations could be used to improve the sensitivity of sedimentation as reported by Calvani et al. [35], though these are more time consuming, especially when processing large numbers of field samples. Most studies exploring the detection limit of the sedimentation test have been experimental, using samples with spiked *Fasciola* spp. eggs, hence, more studies to establish the detection limit of the test in natural infections are required. Other possible reasons for the observed variation in detection rate may be the low sedimentation sensitivity, which failed to detect the low number of eggs in these samples, or possible nonspecific qPCR reactions (with such a difficult matrix as faeces). This is particularly because the qPCR method used in the current study was checked by its creators Alasaad et al. [32] only in a limited range (using for the specificity test only the DNA of *F. magna*, *S. mansoni*, *S. japonicum* and *C. sinensis*).

In the present study, only 3.2% of samples were positive with both qPCR and sedimentation, and coupled with a negative kappa value, this denoted no agreement in the detection of *Fasciola* spp. between the two methods. The present finding was in discord with reports of Calvani et al. [35], who reported a good correlation between the two methods. Arifin et al. [38] also reported poor agreement in the detection of *Fasciola* spp. between PCR and loop-mediated isothermal amplification (LAMP) with sedimentation, though this was considered to be due to the poorer sensitivity of the molecular tests compared to sedimentation. About a quarter (64/277; 23.1%) of the samples that were negative with sedimentation tested positive on qPCR, most likely due to the detection of free worm DNA in faecal samples even in pre-patent infections. Almost a tenth (27/277; 9.7%) of the samples that were positive on sedimentation tested negative with qPCR, possibly due to reduced specificity of the sedimentation test. The discord between the qPCR and sedimentation test in the present study is concerning and could, to the contrary, be suggestive of poor accuracy of the PCR test. Concurrent *Paramphistomum* spp. infections were observed in cattle in the present study and these might have contributed to the lack of specificity of the sedimentation test as the eggs have a similar shape to *Fasciola* eggs; however, the trained eye should be able to discriminate between the two kinds of eggs as they are of different colour [40]. Other reasons for this observation may be related to the limitations of the PCR method that may be due to inhibition, which often occur in the case of stool samples, especially when no internal control was used in the sample tested.

None of the faecal samples tested positive for fluke antigen using the coproELISA. The failure of coproELISA to detect any positive samples in the present study was unexpected given that some samples were positive for *Fasciola* spp. using the sedimentation test. Inability of this technique to detect *Fasciola* spp. can be attributed to the seemingly light infections observed in the present study. Although the coproELISA has ability to detect fluke antigen in faeces prior to completion of the pre-patent period [41], decreased sensitivity was reported with this method in lighter infections of less than 10 EPG, especially in cattle [21,42,43]. The other explanation is with regard to the treatment status of the animals. Animals can return a positive sedimentation test result, but not necessarily have *Fasciola* worms in the bile duct and, therefore, there is no fluke antigen in faeces. Animals with a positive sedimentation result can only be assumed to be infected with *Fasciola* if they have not been treated with fasciolicide within 4 weeks prior to sample collection [41]. However, we did not establish the treatment status of cattle sampled in this study. The original coproELISA [41], based on monoclonal antibody MM3, was shown to be 100% sensitive in the detection of infections in cattle infected with two or more flukes. However, although the commercial coproantigen ELISA is based on the same monoclonal antibody (MM3), the test protocol was substantially modified compared with the initially published assay and data on the sensitivity of the test are scant. Therefore, our findings provide useful information on the suitability of the coproELISA in detection of low faecal egg counts and presumably low worm burdens. The coproELISA may thus be suitable for detecting adult liver fluke infections but is unreliable against immature liver fluke infections [44]. 

It is important to note that most of the positive samples on sedimentation in the present study had low egg counts (< 10 EPG), which most likely affected the detection ability of the coproELISA as reported by Brockwell et al. [20] and Martinez-Sernandez et al. [21]. The current findings support the reports by Novobilský et al. [45], Kajugu et al. [42] and Calvani et al. [22] that the sensitivity of coproELISA is generally low in natural compared to experimental infections, possibly due to the over dilution of the faecal antigens below the detection limit of the test in natural infections. Furthermore, Gordon et al. [43] postulated that, unlike in natural infections, the large infective dose of 200 metacercariae in experimental infections contributes to the increased sensitivity of coproELISA.

The prevalence of bovine fasciolosis observed in the present study was relatively moderate. Mochankana and Robertson [46] reported a similar lower fasciolosis prevalence in communal than commercial cattle, probably due to lack of access of communal cattle to infected drinking water. The 13% prevalence of fasciolosis in the present study as determined by the sedimentation technique was similar to the 16.3% reported in a previous on-station study by Ndhlovu et al. [18] using the ether sedimentation technique. The sedimentation test, thus, is capable of detecting natural *Fasciola* spp. infections and it should not be discarded in antemortem on-farm investigations.

The significant association between detection of *Fasciola* species and age with a greater likelihood of *Fasciola* spp. infection in older compared to young grazing cattle is possibly related to increased length of exposure to infection on pasture in older cattle. This agrees with the findings of Opio et al. [47] and Kouadio et al. [48], who reported a significantly higher *Fasciola* infection rate in cattle aged 4 to 5 years old compared to younger cattle. The present findings are inconsistent with the previous observation of Phiri et al. [49] in Zambia, where younger cattle had a higher prevalence of fasciolosis than older cattle, though post mortem liver inspection and coprological examination were the methods adopted in their study. The reason for this may most likely be due to older cattle developing acquired immunity that resulted in resistance to the flukes. This may also be due to the fact that younger cattle might shed more eggs, while older cattle are more likely to have worms detected in the liver.

Some limitations encountered during this study include the lack of a representative sample from the entire province, relatively small sample size, inability to perform duplicate testing and sometimes inability to morphologically differentiate between *Fasciola* and *Paramphistomum* eggs. Additionally, the coprological Ag ELISA test used was developed for detection of *Fasciola hepatica* and might not accurately detect *F. gigantica* antigens.

## 5. Conclusions

The qPCR was the most sensitive diagnostic test for on-farm detection of bovine fasciolosis followed by sedimentation, while the coproELISA failed to detect *Fasciola* spp. in the current study. There was a relatively moderate (26.4%) prevalence of bovine fasciolosis in communally grazed cattle in the North West Province, South Africa as determined by qPCR. Further studies are required for the characterisation of *Fasciola* spp. using sequencing techniques in communal cattle in South Africa.

## Figures and Tables

**Table 1:** Descriptive analysis and univariate associations between potential animal-level risk factors and *Fasciola* spp. status as determined using the quantitative real-time PCR assay.

Variable (Category)	Number of Positive Cattle (%)	95% CI	*p*-Value
Location			
Makanpastad (*n* = 47)	26 (55.3)	40.12, 69.83	<0.001
Legkolo (*n* = 50)	11 (22.0)	11.53, 35.96	
Makayauna (*n* = 65)	11 (16.9)	8.76, 28.27	
GaMotle (*n* = 60)	8 (13.3)	5.94, 24.59	
Tladistad (*n* = 55)	17 (30.9)	19.14, 44.81	
Breed			
Afrikaner (*n* = 32)	3 (9.4)	1.98, 25.02	0.065
Brahman (*n* = 90)	24 (26.7)	17.89, 37.03	
Crossbreed (*n* = 143)	42 (29.4)	22.06, 37.56	
Sex			
Female (*n* = 246)	62 (25.2)	19.90, 31.11	0.221
Male (*n* = 31)	11 (35.5)	19.23, 54.63	
Age			
2 to 4 years (*n* = 153)	33 (21.6)	15.34, 28.94	0.045
>4 years (*n* = 124)	40 (32.3)	24.15, 41.24	

**Table 2:** Comparison of *Fasciola* quantitative real-time PCR status with consistency of faecal samples collected from cattle in the North West Province, South Africa.

Faecal Consistency	No. of Positive Samples	% of Positive Samples	Odds Ratio	*p*-Value
Good (*n* = 41) (reference)	7	17.1		
Average (*n* = 206)	57	27.7	1.86	0.162
Poor (*n* = 30)	9	30.0	2.08	0.203

**Table 3:** Level of agreement for the detection of *Fasciola* spp. between sedimentation and quantitative real-time PCR.

		qPCR *n* (%)		
		Positive	Negative	Total
**Sedimentation *n* (%)**	Positive	9 (3.2)	27 (9.7)	36 (13.0)
	Negative	64 (23.1)	177 (63.9)	241 (87.0)
	Total	73(26.4)	204 (73.6)	277 (100)

## Data Availability

The data presented in this study are available on request from the corresponding author.
